# Draft Genome Sequence of *Klebsiella pneumoniae* Carbapenemase-Producing *Acinetobacter baumannii* Strain M3AC9-7, Isolated from Puerto Rico

**DOI:** 10.1128/genomeA.00274-15

**Published:** 2015-04-09

**Authors:** Teresa Martínez, Alexander J. Ropelewski, Ricardo González-Mendez, Guillermo J. Vázquez, Iraida E. Robledo

**Affiliations:** a Department of Microbiology and Medical Zoology, University of Puerto Rico, School of Medicine, San Juan, Puerto Rico; b Pittsburgh Supercomputing Center, Carnegie Mellon University, Pittsburgh, Pennsylvania, USA; c Department of Radiological Sciences, University of Puerto Rico, School of Medicine, San Juan, Puerto Rico

## Abstract

We report the draft genome of a multidrug resistant, *Klebsiella pneumoniae* carbapenemase (KPC)-producing *Acinetobacter baumannii* strain M3AC9-7 that belongs to the novel sequence type, ST250. The draft genome consists of a total length of 4.09 Mbp and a G+C content of 38.95%.

## GENOME ANNOUNCEMENT

*Acinetobacter baumannii* is an important opportunistic pathogen associated with worldwide outbreaks in intensive care units and long-term care facilities. *A. baumannii* is responsible for a variety of human infections such as ventilator associated pneumonia, secondary meningitis, bloodstream, wound and urinary tract infections, among others (1). Treatment of *A. baumannii* is challenging due to its multiple intrinsic and acquired mechanisms of antibiotics resistance. The carbapenems are the antibiotics of choice for the treatment of infections caused by multidrug resistant *A. baumannii*, however, their overuse has led to significant antimicrobial resistance in this organism. The production of beta-lactamases, which are mostly encoded in mobile DNA elements, are the most common mode of carbapenem resistance.

In this report, we present the draft genome of a multidrug resistant, *Klebsiella pneumoniae* carbapenemase (KPC)-producing *A. baumannii* clinical isolate, strain M3AC9-7 from Puerto Rico, with the novel sequence type, ST250 (2, 3). Strain M3AC9-7 was isolated in 2009, from the blood cultures of a 58-year-old female hospitalized in an intensive care unit (2). Whole-genome sequencing was performed using an Illumina MiSeq platform in a 2 × 250 bp paired end (PE) configuration by Genewiz, Inc. *De novo* assembly and genome annotation was performed using the Pittsburgh Supercomputing Center Blacklight supercomputer system (4). *De novo* assembly was done using Velvet (version 1.2.10) (5) and additional scaffolding was performed using SSPACE Basic 2.0 (6). The draft genome of *A. baumannii* M3AC9-7 strain consists of 83 contigs, with a total length of 4,091,847 bp, a mean contig length of 49,299 bp, a maximum contig length of 254,812 bp, and a *N*_50_ of 98,350 bp. The G+C content was determined to be 38.95%.

Open reading frames (ORFs) were predicted using Prodigal (version 2.60) (7) and 3,947 ORFs were identified. Genome assembly was annotated by the National Center for Biotechnology Information (NCBI) Prokaryotic Genomes Annotation Pipeline and was corroborated using the complete proteome of *A. baumannii* ATCC 17978 available on UniProtKB/Swiss-Prot (8) and 3,462 ORFs with *E* values of ≤1*e* − 5 were common to both genomes. ORFs that did not align (485 ORFs) were annotated using UniProtKB/TrEMBL (8). Pfam database (9) gave 2,286 unique protein families. tRNA genes and rRNA genes were predicted using tRNAscan-SE (10) and RNAmmer (11), respectively. A total of 64 tRNA genes and 8 rRNA (5S, *n* = 5; 16S, *n* = 1; 23S *n* = 1) were observed.

### Nucleotide sequence accession numbers.

This whole-genome shotgun project has been deposited at DDBJ/EMBL/GenBank under accession no. JTEC00000000. The version described in this paper is JTEC00000000.1.
